# Dihydroartemisinin Alleviates Neuronal Damage and Seizures in Epileptic Mice by Inhibiting Ferroptosis via the SIRT1/FOXO1/SLC7A11/GPX4 Pathway

**DOI:** 10.1002/cns.70798

**Published:** 2026-02-20

**Authors:** Zhipeng You, Cong Huang, Xiaoying Gao, Zhijie Fan, Fan Wei, Yunmin He, Shiyi Zhao, Jiahang Sun

**Affiliations:** ^1^ Department of Neurosurgery The First Affiliated Hospital of Harbin Medical University Harbin China; ^2^ Department of Neurosurgery The Second Affiliated Hospital of Harbin Medical University Harbin China; ^3^ Department of Anesthesiology The Fourth Affiliated Hospital of Harbin Medical University Harbin China

**Keywords:** dihydroartemisinin, epilepsy, ferroptosis, FOXO1, SIRT1, SLC7A11

## Abstract

**Background:**

Epilepsy represents a prevalent neurological disorder. Currently, ferroptosis has been reported to be intricately linked to epilepsy onset and progression. Dihydroartemisinin (DHA) can inhibit the level of ferroptosis in various diseases. Therefore, the present study investigated whether DHA could inhibit seizures and display neuroprotective impacts by impeding ferroptosis.

**Methods:**

In the KA‐induced epileptic mouse model, the effects of DHA on epileptic behavior, cognitive function, and hippocampal neuronal damage were observed. Using both in vivo and in vitro models, the impact of DHA on neuronal injury and ferroptosis‐related markers was investigated. Techniques including molecular docking, Western Blot, immunofluorescence, and CHIP‐qPCR were utilized to analyze the regulatory mechanism of DHA on ferroptosis in epilepsy. Finally, brain tissue samples from patients with temporal lobe epilepsy (TLE) were collected to validate the expression of ferroptosis‐related markers.

**Results:**

Our experimental results showed that DHA attenuated seizures, hippocampal neuronal damage, and memory and learning deficits in epileptic mice. Moreover, DHA inhibited ferroptosis by activating solute carrier family 7 member 11 (SLC7A11) and glutathione peroxidase 4 (GPX4) expression in vivo and in vitro. Subsequently, we found that DHA activated SIRT1 expression in the mouse hippocampus, leading to a decrease in the acetylation level of forkhead box O1 (FOXO1), thereby increasing the transcriptional activity of SLC7A11. Finally, our findings provide preliminary clinical support for the association between ferroptosis and TLE.

**Conclusion:**

In summary, our findings indicate that DHA may have antiepileptic and neuroprotective benefits by suppressing ferroptosis through the SIRT1/FOXO1/SLC7A11/GPX4 signaling pathway.

## Graphical abstract

Dihydroartemisinin (DHA) is an artemisinin derivative. DHA can alleviate ferroptosis in epileptic mice by activating SIRT1 expression, reducing the acetylation level of FOXO1, and thereby enhancing the transcriptional activity of SLC7A11. DHA shows promise as a new treatment for epilepsy.

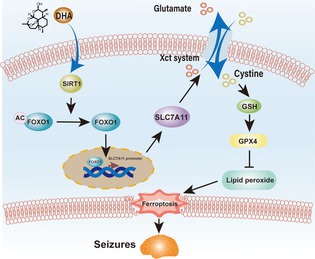

## 1 Background

Epilepsy corresponds to a neurological condition marked by repeated spontaneous seizures, affecting about 70 million individuals globally [1, 2]. Recurrent seizures cause hippocampal neuronal death, impairing brain function, leading to memory loss and cognitive dysfunction, which seriously affects patients' quality of life [3, 4]. Despite the usage of over two dozen antiepileptic medications in clinical settings, approximately 30% of patients still fail to achieve effective seizure control [5, 6, 7]. Among them, the most common one is temporal lobe epilepsy (TLE), which brings serious economic and psychological burdens to patients and their families [8]. Accordingly, developing new antiepileptic drugs is urgent.

Ferroptosis is a newly identified regulated cell death process defined by lipid peroxidation and iron‐dependent reactive oxygen species (ROS) accumulation [9, 10, 11]. Studies have shown that ferroptosis is involved in the pathology and physiology of neurological disorders such as subarachnoid hemorrhage, Alzheimer's disease, stroke, and Parkinson's disease [12, 13, 14, 15, 16, 17]. The presence of ferroptosis has been demonstrated in the epileptic mice hippocampus [10]. Several studies have shown that inhibition of neuronal ferroptosis attenuates seizures and reduces neuronal damage in epileptic mice [18, 19, 20]. This suggests that ferroptosis‐mediated pathological changes are essential in epilepsy onset and progression. Therefore, controlling neuronal ferroptosis occurrence seems to be a promising approach for epilepsy treatment.

Dihydroartemisinin (DHA), an artemisinin derivative, is frequently used in malaria treatment [21, 22, 23]. Numerous experiments have showcased the possible therapeutic value of DHA in malignancies, neurological disorders, and immune system disorders [24, 25, 26, 27, 28]. In diseases such as glioma, leukemia, and lung cancer, DHA has been found to modulate ferroptosis [29, 30, 31]. In addition, DHA has been manifested to enhance memory and cognitive deficits in Alzheimer's disease [32, 33, 34]. DHA also protects neurons from damage in ischemic–hypoxic brain injury [26]. This evidence suggests that DHA is a promising therapeutic approach. Nonetheless, research concerning the neuroprotective properties of DHA in epilepsy through the inhibition of ferroptosis remains limited. This study verified that DHA can alleviate ferroptosis in epilepsy and improve the epileptic phenotype and cognitive dysfunction in mice. We also found that DHA can activate the expression of SIRT1, thereby reducing the acetylation level of FOXO1, enhancing the transcriptional activity of FOXO1 on SLC7A11, and ultimately alleviating ferroptosis in epilepsy. Furthermore, our experiments have preliminarily demonstrated a link between TLE and ferroptosis. In conclusion, our study demonstrates that DHA exerts antiepileptic and neuroprotective effects by impeding ferroptosis through SIRT1/FOXO1/SLC7A11/GPX4 signaling pathway activation. These research results not only help to clarify the pathogenesis of epilepsy, but also are expected to provide new options for the treatment of epilepsy, especially drug‐resistant epilepsy.

## 2 Materials and Methods

### 2.1 Human Tissues

Brain tissues of TLE patients and control patients were obtained from the department of Neurosurgery, the Second Affiliated Hospital of Harbin Medical University, and written consent was obtained from the patients and their families. The study was conducted after obtaining approval from the Second Hospital of Harbin Medical University Ethics Committee in compliance with the Declaration of Helsinki (approval number: KY2024‐036).

### 2.2 Animals

Male C57BL/6J mice (8 weeks, 20–30 g) were purchased from the Liaoning Changsheng Biological Co. Ltd. The C57BL/6J male mouse strain is widely used and well‐documented in epilepsy research and in models of seizure induction. Many prior studies investigating the same or similar models have consistently utilized male mice to ensure comparability and reproducibility of results [8, 35, 36]. Female mice are subject to estrous cycle‐related hormonal fluctuations, which can significantly affect seizure thresholds and pharmacological responses [37, 38]. While these variations are of great biological importance, their presence in the current study design could have introduced additional variability that might confound the interpretation of treatment‐specific effects. Our primary goal in this work was to investigate the treatment efficacy within a well‐established and widely accepted model.

The animals were maintained in specified pathogen‐free environments with constant temperature and humidity, and unrestricted food and drink access. The Second Affiliated Hospital of Harbin Medical University Ethics Committee authorized animal experiments (approval number: KY2018‐108).

### 2.3 In Vivo Experiments

There were two parts to the animal experiment. The animals were randomly divided into four groups in the first part: (1) control group, (2) kainic acid (KA) group, (3) KA + ferrostatin‐1 (Fer‐1) group, and (4) KA + DHA group. For mice in the KA + Fer‐1, Fer‐1 (3 mg/kg, HY‐100579, MCE, USA) was injected intraperitoneally, once a day for 30 days, which was consistent with the dosage of the drugs used in a previous study [39]. For mice in the KA + DHA, DHA (20 mg/kg, HY‐N0176, MCE, USA) was injected intraperitoneally, once a day for 30 days, which was consistent with the dosage of the drugs used in previous studies [34]. Half an hour after the first injection of Fer‐1 or DHA, mice were induced into status epilepticus (SE) via intraperitoneal injection of KA (20 mg/kg, 420318, Sigma, USA) [39]. The control group mice were injected with an identical volume of DMSO.

In the second part of the animal experiment, mice were randomly divided into four groups: (1) control group; (2) kainic acid (KA) group; (3) KA + DHA group; and (4) KA + DHA + EX‐527 group. KA and DHA were used as in the previous phase of the experiment. For mice in the KA + DHA + EX‐527 group, EX‐527 (2 mg/kg, HY‐15452, MCE, USA) was injected intraperitoneally 1 h before KA‐induced SE. DHA was injected intraperitoneally half an hour before KA‐induced SE, once daily for 30 days. EX‐527 was administered once a day for 30 days [40]. For mice in the control, the KA and the KA + DHA groups were injected with an identical volume of DMSO.

All mice were intraperitoneally injected with diazepam (10 mg/kg) 2 h after using KA, and the seizures were terminated [4].

### 2.4 Behavioral Observations

Following the KA injection, the mice were subjected to video monitoring for 30 consecutive days, during which the level of seizures was documented. The mice's behavior was evaluated using the Racine classification: stage 0, no abnormal reaction; stage 1, facial clonus and rhythmic chewing movements; stage 2, rhythmic nodding or wet dog‐like shaking; stage 3, unilateral forelimb clonus; stage 4, forelimb clonic convulsions with rearing; stage 5, generalized clonic convulsions, loss of balance, and falling. Animals in stage IV or higher were considered to have seizures [41]. Two trained experimenters with no knowledge of the experimental protocol performed all behavioral observations.

### 2.5 Cell Culture and Drugs

HT22 cells (Procell Life Science & Technology Co. Ltd., Wuhan, China) were cultured in Dulbecco's modified Eagle's (DMEM, D6429, Sigma, USA) that contained 10% fetal bovine serum at 37°C in a 5% CO_2_.

We employed glutamate (Glu, G8415, Sigma, USA) to stimulate HT22 cells, thereby simulating an in vitro epilepsy model [39]. The in vitro experiments were also divided into two parts. The first part of the cell experiment was divided into four groups: (1) control group; (2) Glu: 5 mM Glu; (3) Glu + Fer‐1: 5 mM Glu + 5 μM Fer‐1; and (4) KA + DHA group: 5 mM Glu + 10 μM DHA. All groups of cell culture media contained the same amount of DMSO. The second part of the cell experiment was also divided into four groups: (1) control group; (2) Glu group: 5 mM Glu; (3) KA + DHA: 5 mM Glu + 10 μM DHA; and (4) Glu + DHA + EX‐527: 5 mM Glu + 10 μM DHA + 10 μM EX‐527. The same amount of DMSO was used in all groups of cell culture media. For the experiments, all cells were treated for 24 h.

### 2.6 Western Blot (WB)

Tissues and cells were processed with RIPA lysis buffer (P0013B, Beyotime, Shanghai, China) to extract total proteins. And nuclear proteins utilizing a Cytoplasmic and Nuclear Protein Extraction Kit (78835, Thermofisher, USA). Electrophoresis was performed using a 10% SDS polyacrylamide gel followed by membrane transfer. After membrane transfer, the membrane was blocked with 5% skimmed milk powder for 1 h, followed by overnight incubation with primary antibody. The next day, the membrane was washed three times with TBST, and the target bands were detected by ECL after secondary antibody incubation. Detailed antibody information is presented in Table S1.

### 2.7 Immunofluorescence Staining (IF)

Brain tissue sections were exposed to deparaffinization and dehydration. Antigen retrieval was carried out using a 1× citrate buffer at elevated temperatures. The sections were closed at room temperature for 1 h. After rinsing the sections with PBS, the primary antibody is incubated overnight, the sections are washed properly the next day and then incubated with the secondary antibody for 1 h at room temperature. Nuclear staining was performed with DAPI (C0065, Solarbio, Beijing, China). Acquisition of fluorescence images was under a fluorescence microscope (Nikon, Tokyo, Japan). Detailed antibody information is presented in Table S1.

### 2.8 Nissl Staining

Brain sections were exposed to dewaxing, rehydrating, and staining with cresyl violet stain (G1430, Solarbio, Beijing, China) for 20 min. The portions were cleaned, dehydrated, clarified, and sealed in distilled water. Acquisition of fluorescence images was performed under a fluorescence microscope (Nikon, Tokyo, Japan).

### 2.9 Transmission Electron Microscopy (TEM)

Tissue fixation was performed using 2.5% glutaraldehyde (G1102, Servicebio, Wuhan, China). Samples were cut after dehydration and stained with 2% uranyl acetate alongside 0.04% lead citrate on a 200‐mesh copper grid before observing mitochondrial properties by TEM (Hitachi, Tokyo, Japan).

### 2.10 Morris Water Maze (MWM) Test

A MWM test was deployed for the assessment of cognitive function in mice [42]. Mice were allocated to swim freely in the maze for 90 s, documenting the duration to locate the platform and permitting the mice to remain on the platform for 30 s. In case of failing to locate the platform within a minute, the mice were directed to locate it. The mice had training for 5 consecutive days. On the 6th day, the platform was removed and the total number of crossings and the duration spent in the target quadrant within 60 s.

### 2.11 Cell Viability Assay

Cell viability was evaluated by the Cell Counting Kit‐8 (CCK‐8) assay (C0037, Beyotime, Shanghai, China). Cells were cultured in a 96‐well plate (5 × 10^3^ cells/well) and then incubated for 30 min with 10 μL of working solution in each well. Finally, cell viability was assessed using a microplate reader at 450 nm.

### 2.12 Ferrous Ion (Fe^2+^) Content Assay

Using a ferrous ion content assay kit (BC5415, Solarbio, Beijing, China) measure Fe^2+^ content assay in hippocampus and cells.

### 2.13 4‐Hydroxynonenal (4HNE) Assay

Detect 4HNE levels using a commercial 4HNE enzyme‐linked immunosorbent assay (ELISA) kit (Zhen Ke Biological Technology Co. Ltd., Shanghai, China) was used following the manufacturer's instructions.

### 2.14 Measurement of Glutathione (GSH), Malondialdehyde (MDA), and Superoxide Dismutase (SOD) Levels

Using GSH and GSSG Assay Kit (S0053, Beyotime, Shanghai, China), Lipid Peroxidation MDA Assay Kit (S0131S, Beyotime, Shanghai, China), and Superoxide Dismutase Activity Assay Kit (BC5165, Solarbio, Beijing, China) to measure GSH, MDA, and SOD levels in hippocampus and cells.

### 2.15 ROS Quantification

Total ROS levels in mouse brain tissue were assayed using dihydroethidium (DHE, S0063, Beyotime, Shanghai, China). Briefly, 10 μM DHE solution was dropped on frozen sections and incubated at 37°C for 30 min, and then washed with PBS. To detect the total ROS levels in cells, they were co‐incubated with 10 μM DHE and complete medium at 37°C for 30 min, followed by appropriate washing with PBS. Then, images were taken under a fluorescence microscope.

### 2.16 Quantitative Real‐Time Reverse Transcription PCR (qRT‐PCR)

Total RNA from hippocampus was extracted with Trizol reagent (15596026CN, Invitrogen, USA). The cDNA was obtained by reverse transcription using a kit (AT311, TransGen Biotech, Beijing, China). Finally, RT‐qPCR was performed through the Top Green qPCR SuperMix kit (AQ601, TransGenBiotech, Beijing, China). The primer information is available at Table S2.

### 2.17 Molecular Docking

A search of the UniProt database yielded the protein sequence. The protein's structure was optimized for energy using Rosetta Relax and modeled with AlphaFold2 since no good resolved structure for molecular docking is available. Using Pymol software, structural preparations like dehydrogenation and hydrogenation were applied to the 3D protein model. The compound structures were sourced by accessing the PubChem database (https://pubchem.ncbi.nlm.nih.gov/). The docking process was carried out via blind docking on the CB‐DOCK2 web server (https://cadd.labshare.cn/cb‐dock2/php/blinddock.php) [43]. Employing Autodock Vina and artificial neural networks, CB‐DOCK2 performed cavity probing. Pymol was used to display the ligand‐receptor 3D conformation, and the PLIP internet service (https://plip‐tool.biotec.tu‐Dresden.de/plip‐web) was relied upon for ligand‐receptor force analysis.

### 2.18 Chromatin Immunoprecipitation (CHIP)

The CHIP followed the guidelines of the Sonication ChIP Kit (RK20258, Abclonal, Wuhan, China). Fresh hippocampus tissue was initially crosslinked with 1% formaldehyde, subjected to ultrasonic fragmentation, and co‐incubated with anti‐FOXO1 antibody and lysate. The chromatin was ultimately eluted and de‐crosslinked, followed by DNA purification utilizing a DNA purification kit (RK30100, Abclonal, Wuhan, China). RT‐qPCR was employed to provide a quantitative assessment of the experimental outcomes. The primer sequences for SCL7A11 were as follows: forward 5′‐AGCTGTGTATGTGAATAGGGTGT‐3′, reverse 5′‐GGAGATAGAAGTGTTTACTCTGAGC‐3′.

### 2.19 Statistical Analysis

Statistical analyses were conducted with GraphPad Prism 8.0 software, and data were presented as mean ± standard error of the mean (SEM). Normality tests were performed on all quantitative data using the Shapiro Wilk test or the Kolmogorov Smirnov test. Student's *t*‐test was utilized for comparisons between two groups. For differences in variables among more than two groups, one‐way analysis of variance (ANOVA) or two‐way ANOVA was employed, trailed by Tukey's post hoc test. *p* < 0.05 was considered statistically significant.

## 3 Results

### 3.1 DHA Alleviates Epileptic Phenotype and Cognitive Dysfunction in KA‐Induced Mice

**FIGURE 1:**
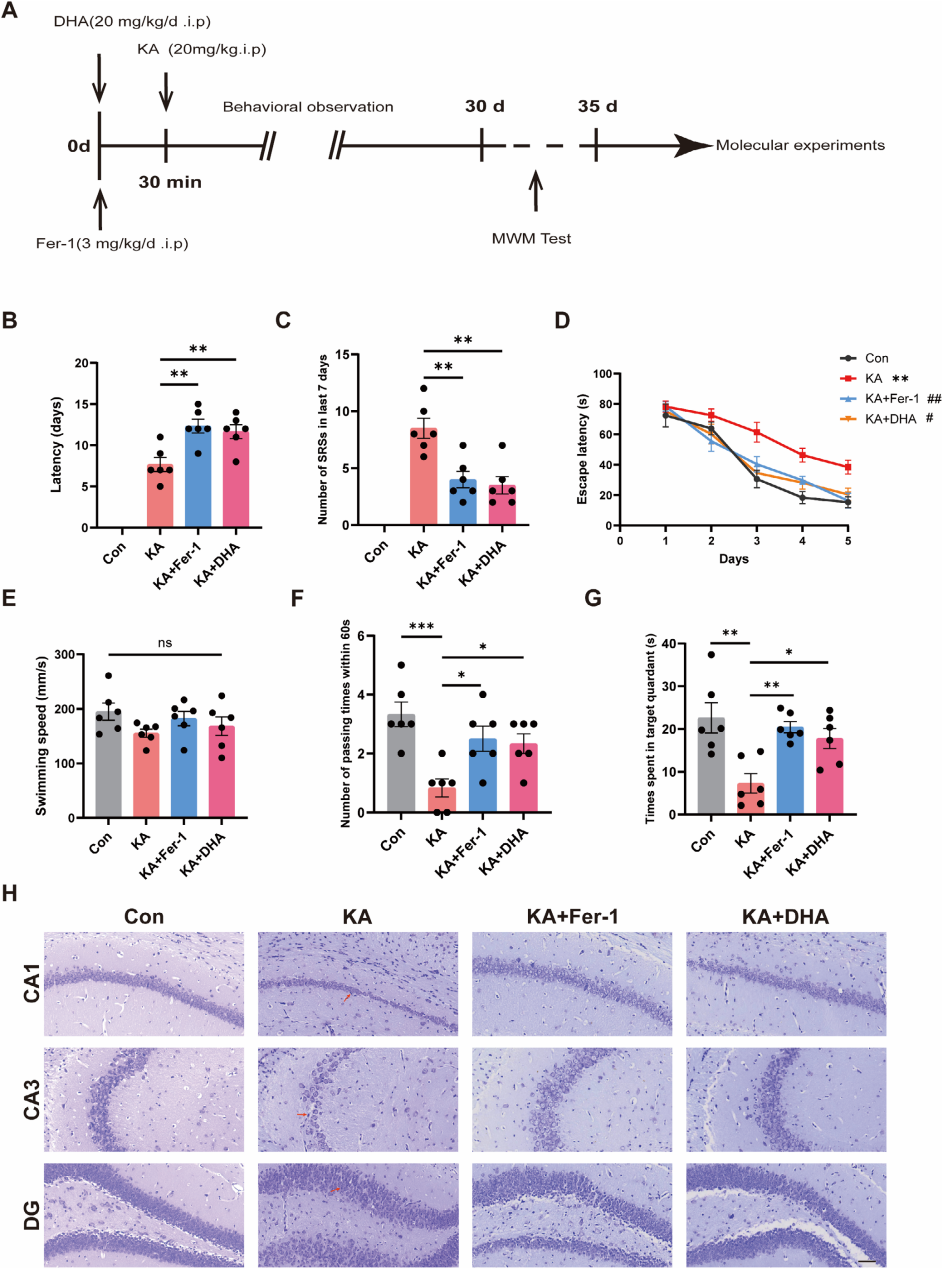
DHA mitigates epileptic symptoms and cognitive impairment in KA‐induced mice. (A) An illustration of the experimental design. (B) The latency period of SRSs with DHA or Fer‐1 (*n* = 6). (C) The number of SRSs in mice during the last 7 days of behavioral observation with DHA or Fer‐1 (*n* = 6). (D) Trends in escape latency across training days for each group (*n* = 6). **p < 0.01, the control group; ^*##*^ *p* < 0.01, compared with the KA group; ^*#*^ *p* < 0.05, compared with the KA group. (E) Comparison of swimming speed per group (*n* = 6). (F) Platform crossings per group (*n* = 6). (G) Duration in the target quadrant per group (*n* = 6). (H) Representative Nissl staining images of CA1, CA3, and DG regions in mouse brains (The red arrows indicates damaged neurons). The scale bar = 50 μm. ***p < 0.001, **p < 0.01, *p < 0.05.

To verify the role of DHA in epilepsy, we observed behavioral changes in KA‐induced mice. The experimental procedure is described in (Figure 1A). We found that Fer‐1 or DHA administration prolonged the latency period of spontaneous recurrent seizures (SRSs) compared with the KA group mice, and the number of SRS in the KA + Fer‐1 group and KA + DHA group was significantly lower than that in the KA group mice during the last 7 days of behavioral observation (Figure 1B,C). Next, we examined DHA's impact on cognitive dysfunction in mice with KA‐induced epilepsy using the MWM test. During the first 5 days of testing, we recorded the escape latency of each group of mice. Notably, the escape latency of KA mice was significantly prolonged, unlike the control. In contrast, the escape latency in DHA or Fer‐1‐treated mice was considerably shorter than in KA mice (Figure 1D). Simultaneously, there was no notable disparity in the swimming velocity of the mice among the groups (Figure 1E). This indicates normal motor function of the mice. On the sixth day of testing, we found that the number of times the KA mice traversed the platform and spent less time in the target quadrant compared to the control mice. However, the DHA or Fer‐1‐treated mice traversed the platform more often and remained in the target quadrant for a longer time compared to the KA mice (Figure 1F,G). The representative trajectories of each group of mice during the probe trial can be observed in Figure S1. This suggests to us that DHA may improve learning as well as memory deficits in epileptic mice.

We further explored the possible neuroprotective effects of DHA in epileptic mice using Nissl Staining. The results show a tendency to massive neuronal loss in the KA group of mice compared with control mice in the CA1, CA3, and DG regions, which was reversed by DHA or Fer‐1 treatment (Figure 1H, Figure S2). The above results show that DHA alleviates the epileptic phenotype and cognitive dysfunction in KA‐induced mice by inhibiting ferroptosis.

### 3.2 DHA Inhibits Ferroptosis in KA‐Induced Epileptic Mice

**FIGURE 2:**
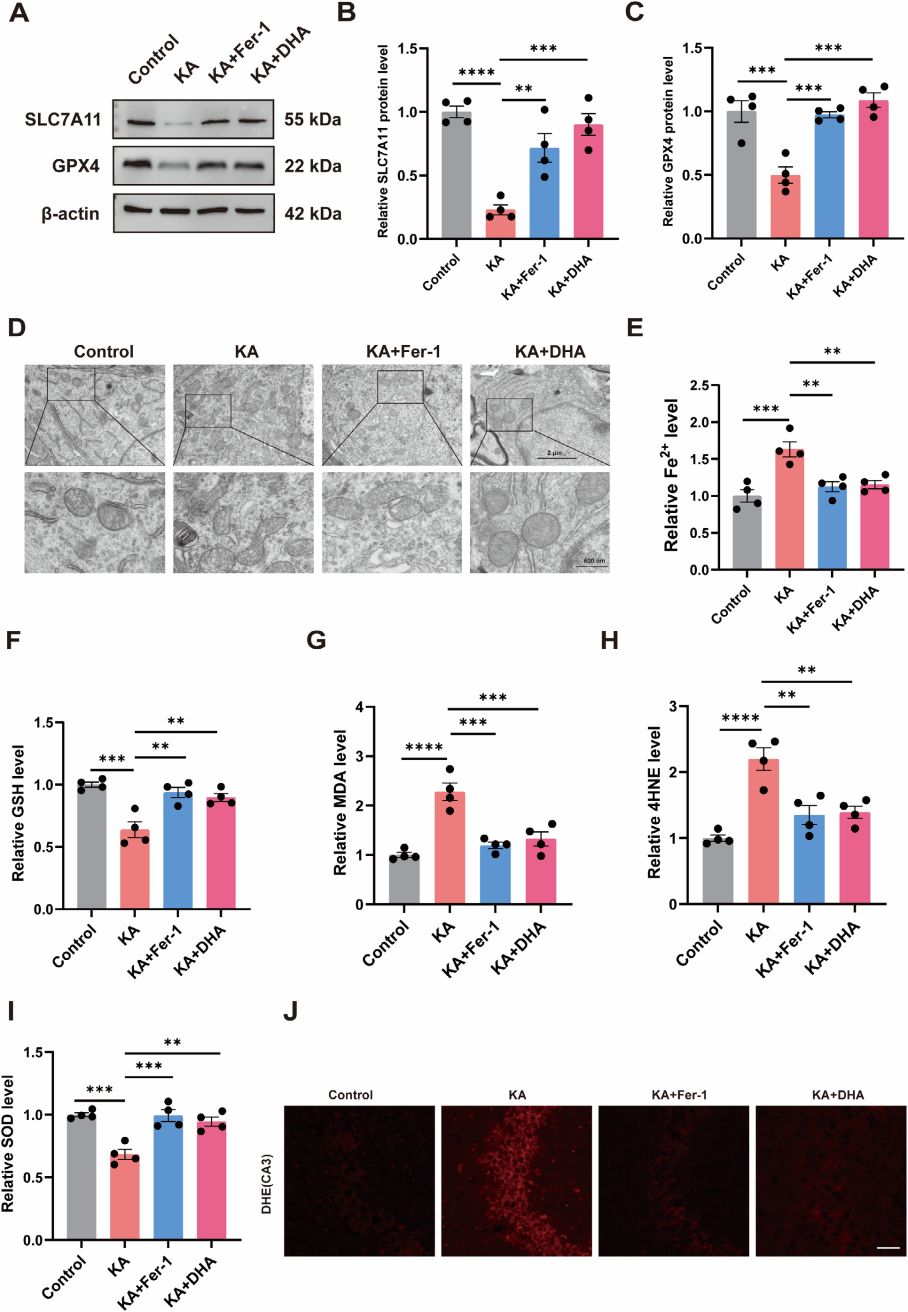
DHA inhibits ferroptosis in KA‐induced epileptic mice. (A–C) Protein expressions of SLC7A11 and GPX4 were detected by Western Blot (*n* = 4). (D) TEM was used to observe mitochondrial morphology in each group. (E) Fe^2+^ levels in mouse hippocampal tissue (*n* = 4). (F–I) GSH, MDA, 4HNE and SOD levels in mouse hippocampal tissues (*n* = 4). (J) Representative images of DHE staining in the CA3 region. The scale bar = 50 μm. ****p < 0.0001, ***p < 0.001, **p < 0.01.

To gain additional insight into the DHA inhibitory effect on ferroptosis in vivo, we analyzed SLC7A11 and GPX4 protein levels in the hippocampus tissue of mice. The hippocampus of KA‐induced epileptic mice experienced diminished SLC7A11 and GPX4 protein expression relative to control mice while administrating DHA or Fer‐1 enhanced SLC7A11 and GPX4 protein expression (Figure 2A–C). The TEM results showed hippocampal neuronal mitochondrial morphology atrophy in KA mice, a characteristic morphological change of ferroptosis. Interestingly, DHA or Fer‐1 treatment significantly attenuated the morphological changes induced by KA (Figure 2D). Meanwhile, we found that the level of Fe^2+^ was significantly elevated in KA‐provoked epileptic mice hippocampus compared to control mice, while the Fe^2+^ level was significantly lower after DHA or Fer‐1 treatment in KA mice (Figure 2E). Furthermore, we assessed GSH, MDA, 4HNE and SOD levels in hippocampus tissue. We found that GSH and SOD levels were decreased, MDA and 4HNE levels were increased in the hippocampus of KA mice in comparison to control mice, and the intervention of DHA or Fer‐1 reversed this trend (Figure 2F–I). Moreover, we found that total ROS levels were significantly elevated in the CA1 and CA3 brain regions of KA mice compared to control mice, while total ROS levels were significantly lower in the hippocampus of DHA or Fer‐1‐treated mice (Figure 2J, Figure S3). These results suggest that DHA inhibits ferroptosis in KA‐induced epileptic mice.

### 3.3 DHA Inhibits Glu‐Induced Ferroptosis in HT22 Cells

**FIGURE 3:**
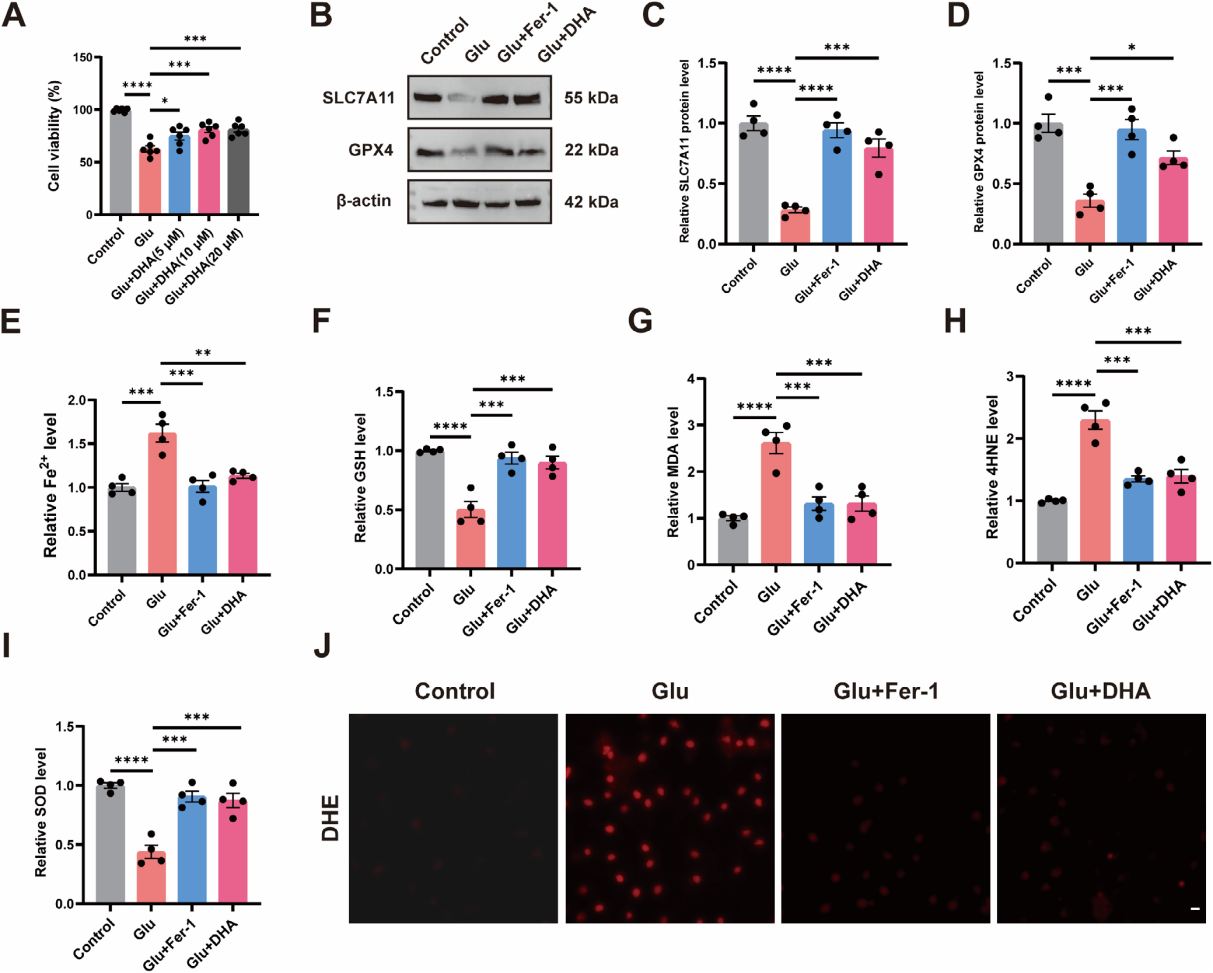
DHA inhibits Glu‐induced ferroptosis in HT22 cells. (A) HT22 cell viability of different concentrations of DHA (*n* = 6). (B–D) Protein expressions of SLC7A11 and GPX4 were detected by Western Blot after Glu, DHA, or Fer‐1 treatment (*n* = 4). (E) Fe^2+^ levels in HT22 cells after Glu, DHA, or Fer‐1 treatment (*n* = 4). (F–I) GSH, MDA, 4HNE and SOD levels in HT22 cells after Glu, DHA, or Fer‐1 treatment (*n* = 4). (J) Representative images of DHE staining in HT22 cells after Glu, DHA, or Fer‐1 treatment. The scale bar = 50 μm. ****p < 0.0001, ***p < 0.001, **p < 0.01, *p < 0.05.

Then, we utilized Glu to induce HT22 cells to mimic an in vitro neuronal injury model and explored the relationship between DHA and ferroptosis in HT22 cells. We first used different concentrations of DHA to treat HT22 cells. The results showed that DHA at a concentration of 10 μM significantly ameliorated the Glu‐induced decrease in cell viability, but this effect was not enhanced with increasing DHA concentration. Therefore, we chose a concentration of 10 μM for the subsequent experiments (Figure 3A). Following that, we assessed the protein levels of SLC7A11 and GPX4 in HT22 cells of each group, revealing significantly diminished SLC7A11 and GPX4 protein levels in Glu‐induced HT22 cells relative to the control, while DHA or Fer‐1 treatment significantly elevated SLC7A11 and GPX4 protein levels compared to the Glu group (Figure 3B–D). In addition, we examined the Fe^2+^ level in HT22 cells, which was significantly elevated after Glu induction in comparison to the control, and using DHA or Fer‐1 reduced the Glu‐induced elevation of the Fe^2+^ level (Figure 3E). Subsequently, we assessed GSH, MDA, 4HNE, and SOD levels in HT22 cells. Consistent with in vivo experiments, compared with the control cells, GSH and SOD levels were decreased, MDA and 4HNE levels were increased in the Glu group, and treatment with DHA or Fer‐1 reversed the alterations in GSH, MDA, 4HNE, and SOD levels (Figure 3F–I). We also found that DHA or Fer‐1 significantly reduced total ROS accumulation by Glu induction in HT22 cells (Figure 3J). This suggests that DHA inhibits Glu‐induced ferroptosis in neuronal cells.

### 3.4 DHA Alleviates Seizures in KA‐Induced Mice by Activating SIRT1

**FIGURE 4:**
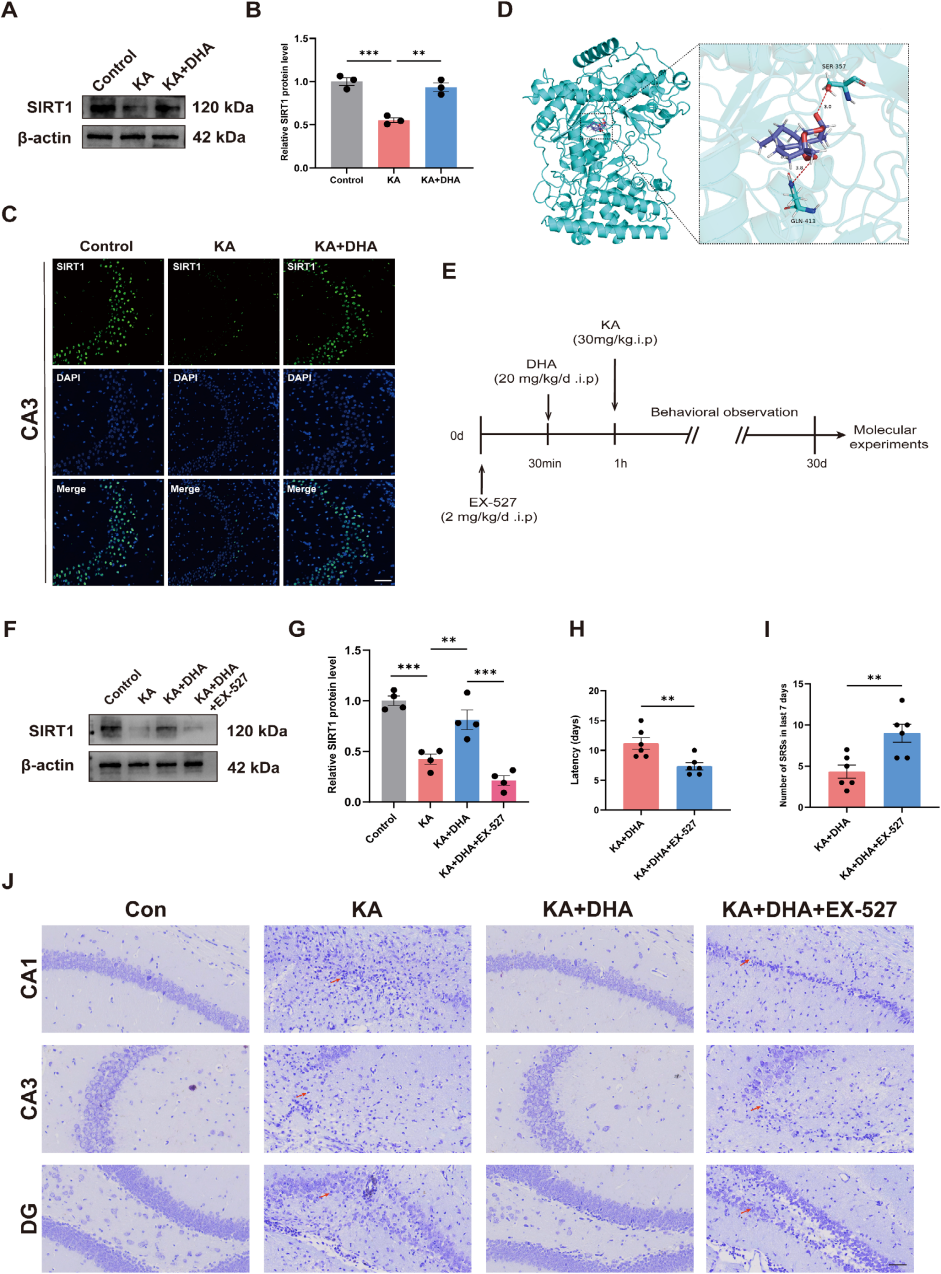
DHA alleviates seizures in KA‐induced mice by activating SIRT1. (A, B) Protein expressions of SIRT1 were detected by Western Blot after DHA injection (*n* = 3). (C) Immunostaining of CA3 regions for SIRT1 after DHA injection. The scale bar = 50 μm. (D) Molecular docking of SIRT1 and DHA. (E) Illustration of the experimental design. (F, G) Protein expressions of SIRT1 were detected by Western Blot after EX‐527 injection (*n* = 4). (H) The latency period of SRSs after EX‐527 injection (*n* = 6). (I) The number of SRSs in mice during the last 7 days of behavioral observation after EX‐527 injection (*n* = 6). (G) Representative Nissl staining images of CA1, CA3, and DG regions in mouse brains after EX‐527 injection (The red arrows indicates damaged neurons). The scale bar = 50 μm. ***p < 0.001, **p < 0.01.

Previous studies have shown that DHA activates SIRT1, thereby ameliorating cerebral ischemia–reperfusion injury in rats [44]. Activating SIRT1 attenuates seizures and reduces neuronal damage [45, 46]. Therefore, we speculated that DHA may attenuate the KA‐induced epileptic phenotype in mice by activating SIRT1. To verify our speculation, we initially investigated the DHA effect on SIRT1 expression using Western Blot and IF, indicating that there was significantly lower SIRT1 expression in the hippocampus of KA mice than in the control; however, SIRT1 expression was significantly elevated after DHA treatment (Figure 4A–C, Figure S4). This suggests that DHA can activate the expression of SIRT1. The molecular docking results revealed specific contacts between the chemical and the protein, with a projected binding energy of −7.3 kcal/mol for the ligand and the acceptor (Figure 4D), indicating a strong binding activity of DHA and SIRT1.

To investigate whether DHA could attenuate KA‐induced seizures in mice by activating SIRT1, we used EX‐527 (a SIRT1 inhibitor) to inhibit SIRT1 activation (Figure 4E–G). Behavioral results showed that co‐treatment with EX‐527 significantly reduced the latency period of SRSs and increased the number of SRSs in mice during the last 7 days of behavioral observation compared with DHA alone in epileptic mice (Figure 4H,I). In addition, we found that EX‐527 blocked the protective effect of DHA on neurons (Figure 4J Figure S5). Taken together, DHA may attenuate seizures and reduce neuronal damage in epileptic mice by activating SIRT1.

### 3.5 DHA Attenuates Ferroptosis in KA‐Induced Epileptic Mice by Activating SIRT1

**FIGURE 5:**
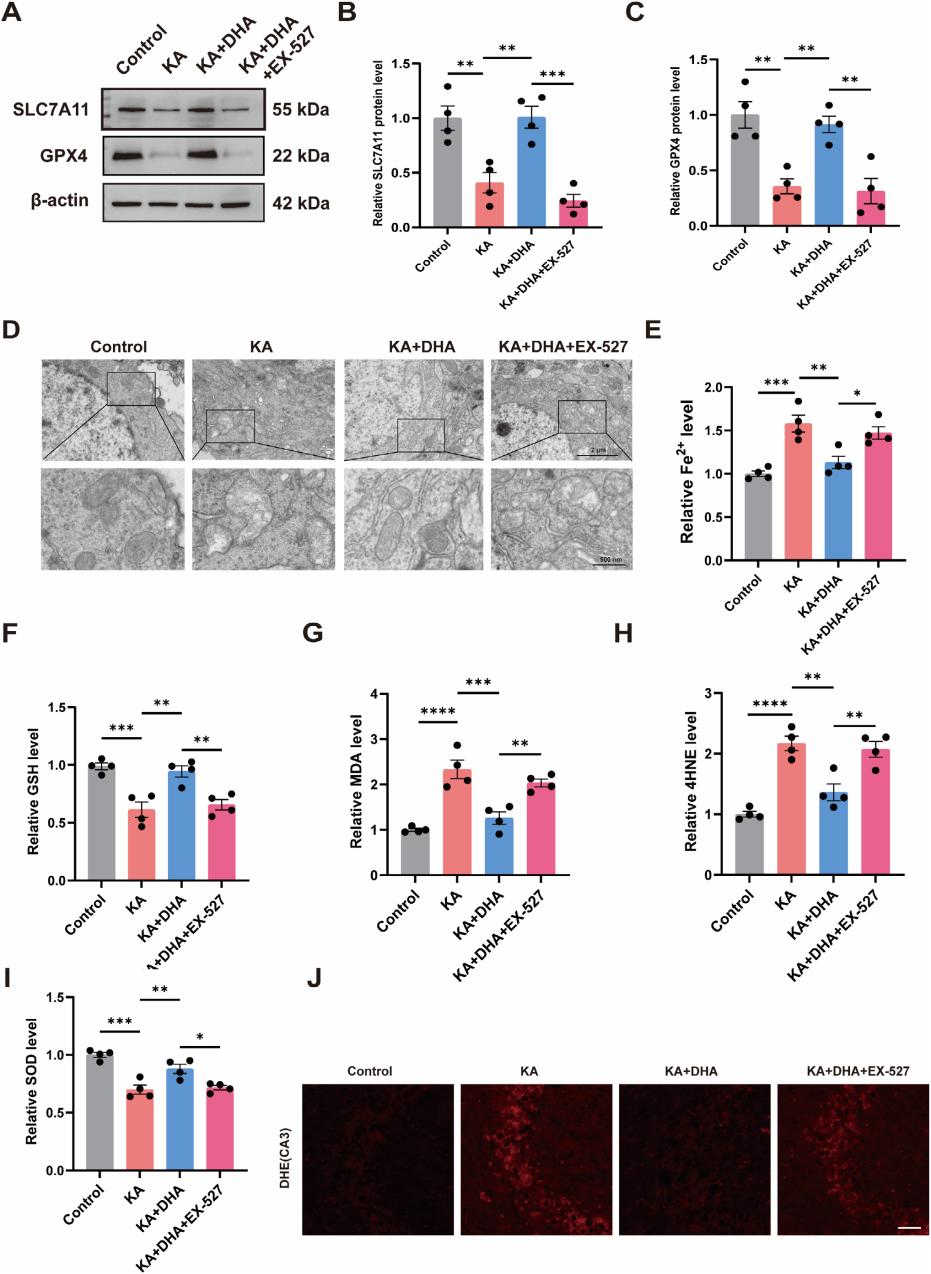
DHA attenuates ferroptosis in KA‐induced epileptic mice by activating SIRT1. (A–C) Protein expressions of SLC7A11 and GPX4 by Western Blot after EX‐527 injection (*n* = 4). (D) TEM was used to observe mitochondrial morphology after EX‐527 injection. (E) Fe^2+^ levels in hippocampus after EX‐527 injection (*n* = 4). (F–I) GSH, MDA, 4HNE and SOD levels in hippocampus after EX‐527 injection (*n* = 4). (J) Representative images of DHE staining in the CA3 region after EX‐527 injection. The scale bar = 50 μm. ****p < 0.0001, ***p < 0.001, **p < 0.01, *p < 0.05.

Activation of SIRT1 inhibits the occurrence of ferroptosis [39]. In the next experiment, we explored whether DHA could attenuate KA‐induced ferroptosis in epileptic mice by activating SIRT1. In the epileptic mice hippocampal tissue, EX‐527 blocked the effect of DHA on the elevation of SLC7A11 and GPX4 proteins (Figure 5A–C). The TEM observed that mitochondrial morphology in hippocampal neurons of mice with the combined intervention of DHA and EX‐527 showed atrophy compared to mice treated with DHA alone (Figure 5D). In addition, Fe^2+^ level in the hippocampus of the KA + DHA + EX‐527 group mice was significantly elevated compared to the KA + DHA group mice (Figure 5E). Detection of GSH, MDA, 4HNE and SOD levels, the results show that in the epileptic mice hippocampus, using EX‐527 blocked the effects of DHA on GSH, MDA, 4HNE and SOD (Figure 5F–I). Finally, we found that the KA + DHA + EX‐527 group mice exhibited higher accumulation of total ROS in CA1 and CA3 brain regions compared with the KA + DHA group mice (Figure 5J, Figure S6). These results suggest that DHA may inhibit KA‐induced ferroptosis by activation of SIRT1 in KA‐induced epileptic mice.

### 3.6 DHA Attenuates Glu‐Induced Ferroptosis in HT22 Cells by Activating SIRT1

**FIGURE 6:**
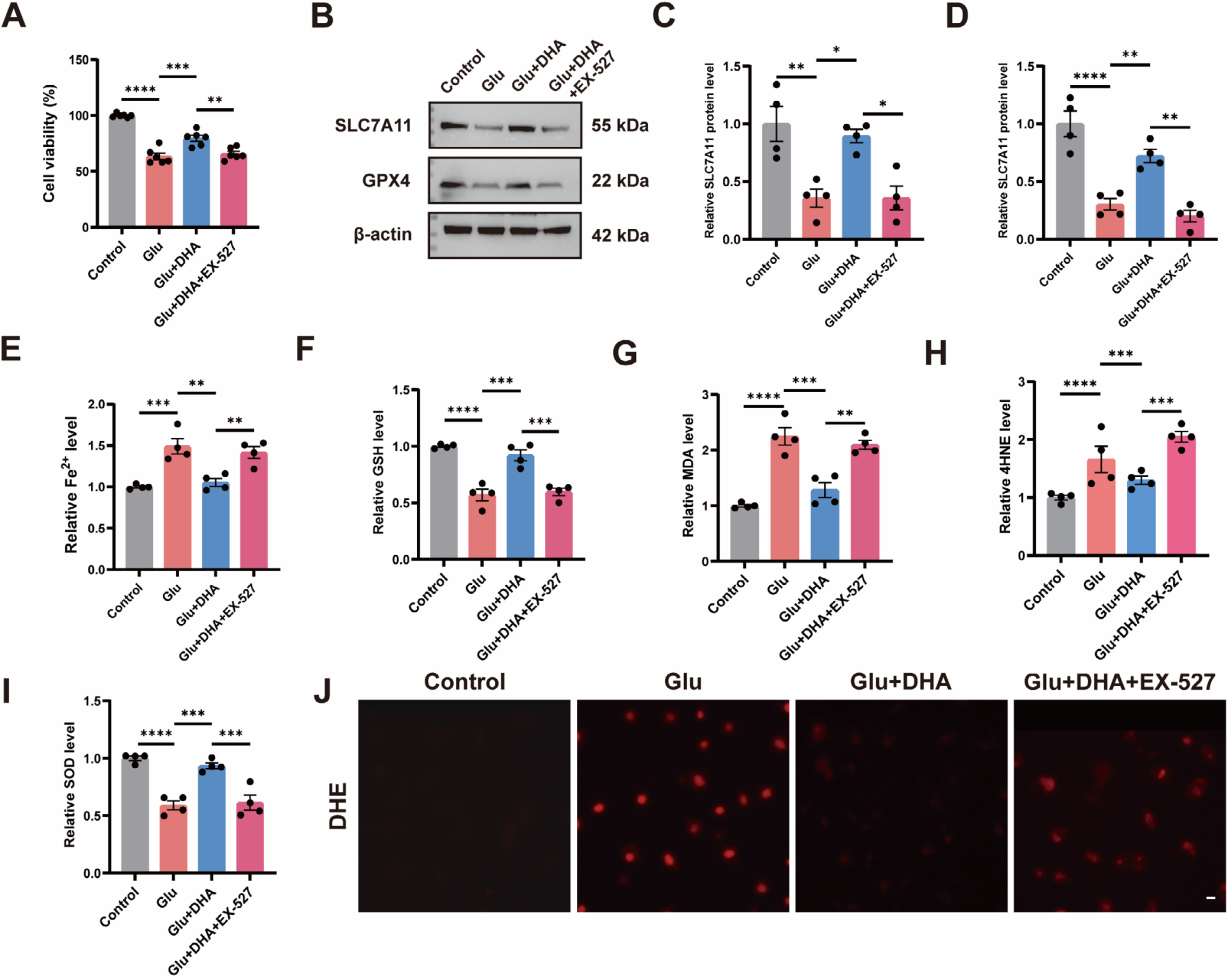
DHA attenuates Glu‐induced ferroptosis in HT22 cells by activating SIRT1. (A) HT22 cell viability after EX‐527 treatment (*n* = 6). (B–D) Protein expressions of SLC7A11 and GPX4 in HT22 cells by Western Blot after EX‐527 treatment (*n* = 4). (E) Fe^2+^ levels in HT22 cells after EX‐527 treatment (*n* = 4). (F–I) GSH, MDA, 4HNE and SOD levels in HT22 cells after EX‐527 treatment (*n* = 4). (J) Representative images of DHE staining in the HT22 cells after EX‐527 treatment. The scale bar = 50 μm. ****p < 0.0001, ***p < 0.001, **p < 0.01, *p < 0.05.

Next, we verified whether DHA could attenuate ferroptosis by activating SIRT1 in HT22 cells. We inhibited SIRT1 expression in HT22 cells using EX‐527 (Figure S7). The CCK8 results showed that cell viability was significantly reduced in the Glu + DHA + EX‐527 group compared to the Glu + DHA group (Figure 6A). Western Blot results showed that EX‐527 also reversed the activation of SLC7A11 and GPX4 proteins by DHA (Figure 6B–D). Compared to the Glu + DHA group, treatment with EX‐527 also increased the Fe^2+^ level in HT22 cells (Figure 6E). In addition, inhibition of SIRT1 expression similarly blocked the effects of DHA on GSH, MDA, 4HNE and SOD in HT22 cells (Figure 6F–I). Finally, compared to the Glu + DHA group, the total ROS levels were significantly increased in the cells of the Glu + DHA + EX‐527 group (Figure 6J). This suggests that DHA contributes to inhibiting ferroptosis in an vitro model of neuronal injury, perhaps through activation of SIRT1.

### 3.7 DHA Promotes SLC7A11 Transcription by Deacetylating FOXO1 Through Activation of SIRT1

**FIGURE 7:**
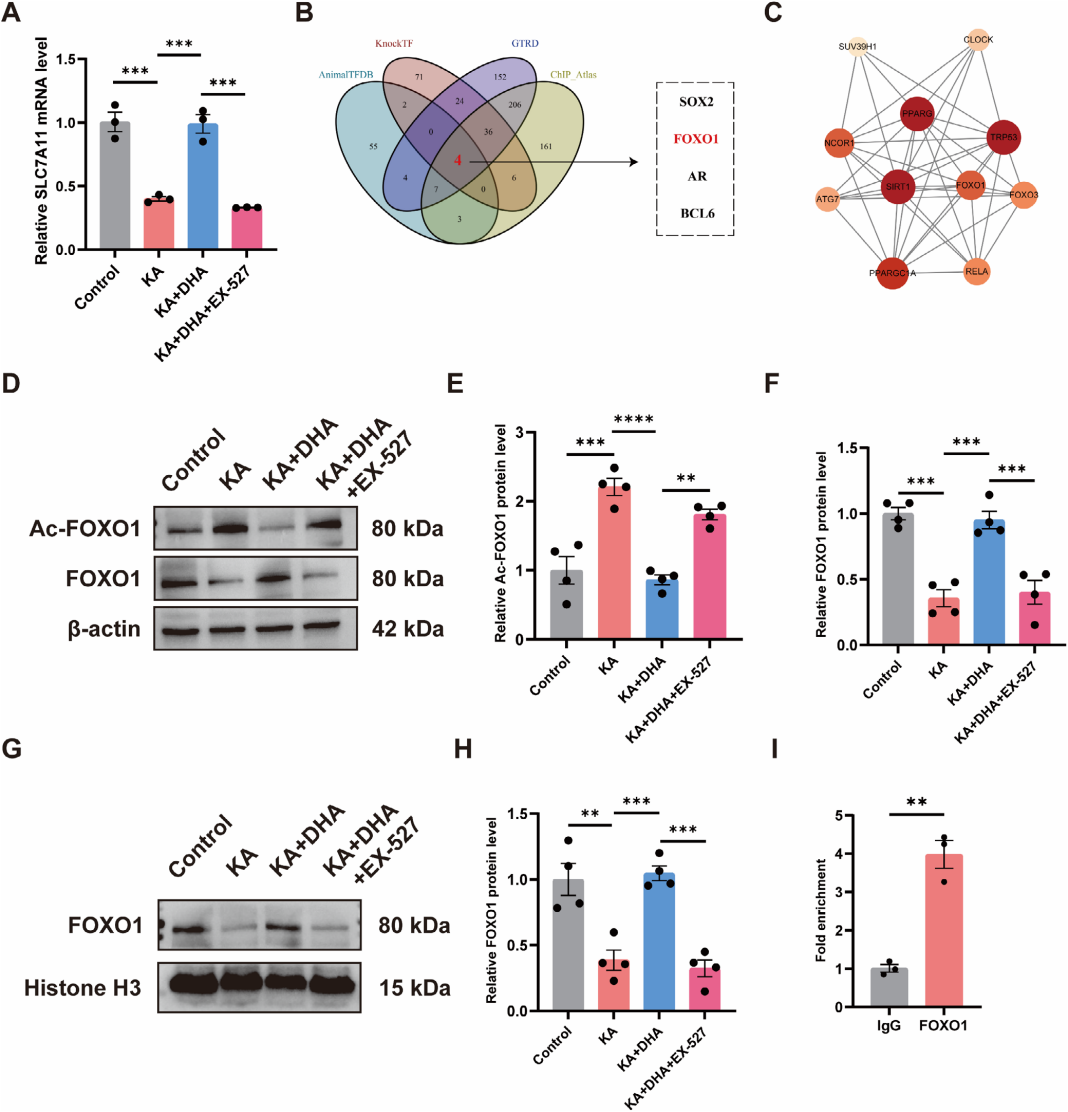
DHA activates SIRT1 to deacetylate FOXO1 to promote SLC7A11 mRNA transcription. (A) SLC7A11 mRNA levels in the hippocampus after EX‐527 treatment (*n* = 3). (B) The overlap of SLC7A11 transcription factors in four databases was shown in a Venn graph. (C) Bubble chart showed the predicted SIRT1 interaction protein. (D–F) Protein expressions of Ac‐FOXO1 and FOXO1 in the hippocampus by Western Blot after EX‐527 treatment (*n* = 4). (G, H) Protein expressions of FOXO1 in the hippocampus nuclear lysate by Western Blot after EX‐527 treatment (*n* = 4). (I) CHIP‐qPCR was used to verify that FOXO1 could bind the SLC7A11 promoter (*n* = 3). ****p < 0.0001, ***p < 0.001, **p < 0.01.

To further investigate the role of DHA in regulating ferroptosis through SIRT1, we examined the mRNA expression of SLC7A11 in the hippocampus of mice. The qRT‐PCR results showed that DHA activated the mRNA levels of SLC7A11, while EX‐527 reversed this trend (Figure 7A). Therefore, we speculate that SIRT1 may alter the transcriptional level of SLC7A11 mRNA by regulating a particular class of transcription factors, thereby changing the transcriptional level of SLC7A11. Subsequently, we used 4 databases, AnimalTFDB, GTRD, KnockTF, and ChIP Atlas, to predict the transcription factors of SLC7A11, and after taking the intersection, the results that there are 4 transcription factors (SOX2, FOXO1, AR, and BCL6) that may bind to the SLC7A11 promoter (Figure 7B). Meanwhile, we predicted the interacting proteins of SIRT1 by STRING online database, and surprisingly, that forkhead box O1 (FOXO1) might interact with SIRT1 (Figure 7C). The SIRT1 is known to be a highly conserved nicotinamide adenine dinucleotide NAD^+^‐dependent histone deacetylase [47, 48]. Studies have shown that acetylation of FOXO1 attenuates the transcriptional level of FOXO1 [49, 50, 51]. Therefore, we speculate that DHA may activate the expression of SIRT1 to deacetylate FOXO1, which ultimately affects the transcriptional regulation of SLC7A11 by FOXO1. To test the hypothesis, we investigated the alterations in the protein levels of FOXO1 and acetylated FOXO1 (Ac‐FOXO1) in the hippocampus of mice. Western Blot results indicated that DHA enhanced FOXO1 expression and reduced the level of Ac‐FOXO1, however, EX‐527 reversed this pattern (Figure 7D–F). Moreover, we examined the FOXO1 protein expression in the hippocampus nuclear lysate, revealing that EX‐527 reduced the nuclear translocation of FOXO1 activated by DHA (Figure 7G,H). Finally, CHIP‐qPCR showed that FOXO1 can bind directly to the SLC7A11 promoter (Figure 7I). In conclusion, these above results suggest that DHA can activate SIRT1 to deacetylate FOXO1 and thereby promote SLC7A11 transcription.

### 3.8 Validation of Ferroptosis in Patients With TLE

**FIGURE 8:**
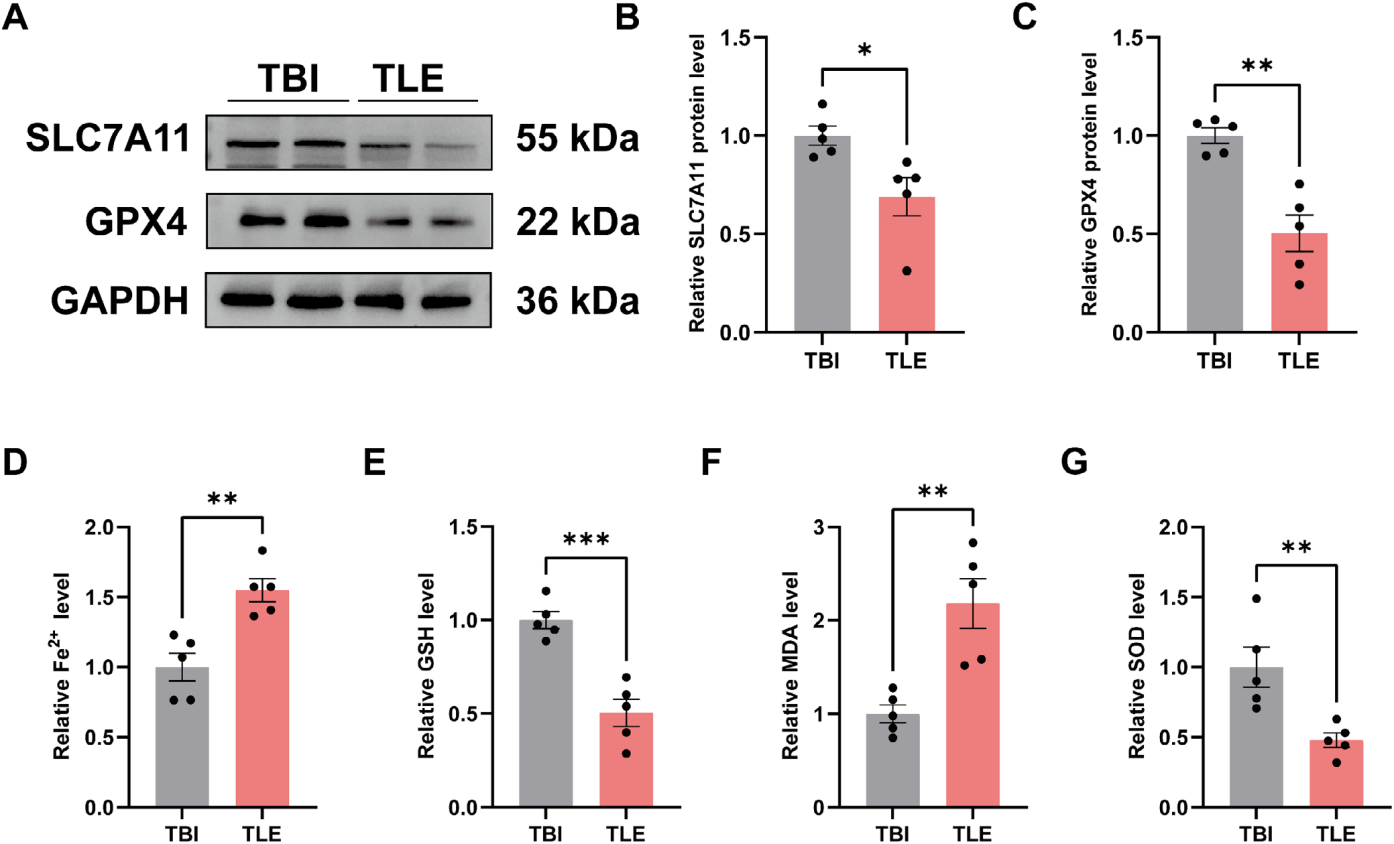
Validation of ferroptosis in TLE patients. (A–C) Protein expressions of SLC7A11 and GPX4 in the brain of TLE and TBI by Western Blot (*n* = 5). (D) Fe^2+^ levels in the brain of TLE and TBI (*n* = 5). (E–G) GSH, MDA, and SOD levels in the brain of TLE and TBI (*n* = 5). ***p < 0.001, **p < 0.01, *p < 0.05.

To confirm the clinical significance of the above results, we assessed the ferroptosis‐related indicators in the surgical tissues of TLE patients. As a control group, we employed traumatic brain injury (TBI) patients (Table S3). We first found no significant differences in age or sex between the two groups (Table S4). The findings indicated that in the brain tissues of TLE patients, SLC7A11 and GPX4 protein expression was diminished, and the Fe^2+^ concentration in the cerebral tissues was significantly increased relative to TBI patients (Figure 8A–D). And our results indicated that TLE patients exhibited diminished GSH and SOD levels and elevated MDA levels (Figure 8E–G). These findings provide preliminary clinical support for the association between ferroptosis and TLE.

## 4 Discussion

Typically, DHA is purified from the natural herb artemisinin and has a unique endoperoxide bridge structure [52] besides being noted for its favorable anticancer properties. Recently, DHA has been elucidated to be crucial in improving cognitive dysfunction and neuroprotection [32, 53]. In the treatment of epilepsy, the hindering effect of the blood–brain barrier to drugs is an issue that needs to be taken into account [54, 55]. The DHA demonstrates significant permeability across the blood–brain barrier, supporting its potential application in epilepsy treatment. Epilepsy is often accompanied by substantial neuronal loss and excessive oxidative stress [56, 57]. In addition, cognitive dysfunction brought about by epilepsy severely affects the patient's quality of life. The results of the behavioral tests showed that DHA had an inhibitory effect on seizures. We examined the impact of DHA on the mice's cognitive function with the MWM test. The DHA‐treated mice significantly improved learning and memory abilities compared with the KA group. The following results of Nissl staining showed that DHA attenuated hippocampal neuronal loss in epileptic mice. Altogether, DHA may be an effective drug for epilepsy treatment. The Fer‐1, an inhibitor of ferroptosis, was also used in our experiments for the above assays, and found that in epileptic mice, intervention with Fer‐1 produced results consistent with those of DHA, suggesting that DHA may exert antiepileptic and neuroprotective effects by inhibiting ferroptosis.

Growing evidence shows that ferroptosis in neurons is closely associated with epilepsy [39, 58, 59]. The L‐cystine/L‐glutamic acid reverse transporter (Xct) system is a vital intracellular antioxidant mechanism that facilitates the transmembrane transport of glutamate as well as cysteine, with SLC7A11 serving as a key component [60, 61, 62]. The Xct system inhibition results in intracellular cystine depletion, which impacts the antioxidant GSH synthesis [63]. The GSH is the predominant reductant in mammalian cells, crucial for iron–sulfur cluster formation, and functions as a cofactor for numerous enzymes, such as GPX. Impaired GSH reduces GPX4 synthesis [64, 65]. The GPX4 is a crucial regulator of ferroptosis, and its absence leads to lipid peroxidation and, ultimately, ferroptosis [66, 67]. It has been shown that by targeting the activation of GPX4, ferroptosis can be inhibited, thereby attenuating seizures in animal models [68]. In the present study, we confirmed the inhibitory impact of DHA on ferroptosis in vivo and in vitro. Our findings indicated that DHA and Fer‐1 administration stimulated SLC7A11 and GPX4 protein expression. Furthermore, DHA and Fer‐1 intervention reduced the intracellular Fe^2+^ level and lipid peroxidation levels. Moreover, we observed that DHA reversed KA‐induced morphological changes in the mitochondria of hippocampal neuronal cells in vivo. These experimental results provide in vivo and in vitro evidence that DHA may inhibit ferroptosis in epilepsy.

SIRT1, a NAD^+^‐dependent deacetylase family member, is crucial in governing cellular senescence, inflammation, and anti‐oxidative stress [69, 70, 71]. Recently, SIRT1 has been found to be an essential target for inhibiting ferroptosis [72, 73, 74, 75]. Edaravone can exert antidepressant impacts by hampering ferroptosis via SIRT1 activation [76]. Resveratrol can also activate SIRT1 to inhibit the occurrence of ferroptosis in heart failure, thereby improving cardiac function [77]. In epilepsy, it has also been shown that activation of SIRT1 inhibits the onset of ferroptosis, thereby attenuating seizures in mice [39]. In our study, DHA activated SIRT1 expression in the hippocampus of epileptic mice, whereas EX‐527 reversed the activating effect of DHA on SIRT1. Moreover, we found that EX‐527 blocked the inhibitory effects of DHA on KA‐induced epileptic phenotype. This suggests that DHA may exert its antiepileptic effects by activating SIRT1. Furthermore, EX‐527 similarly reversed the inhibitory influence of DHA on ferroptosis in epileptic mice. Additionally, inhibiting SIRT1 blocked the inhibitory effect of DHA on ferroptosis in vitro. Therefore, it suggests that in either in vivo or in vitro, DHA can suppress ferroptosis through activating SIRT1. It has been shown that SIRT1 can regulate the deacetylation of various cytoplasmic substrates, thereby regulating transcription factors to control cellular biological processes [77, 78, 79]. In mechanism research, after blocking SIRT1 activity with the SIRT1 inhibitor EX‐527, the upregulation of SLC7A11 and GPX4 by DHA was simultaneously reversed, suggesting that the expression regulation of these two genes depends on the activation of SIRT1. FOXO1 is a transcription factor, and the level of acetylation of FOXO1 negatively correlates with transcriptional activity [51, 80]. In our study, DHA administration increased the deacetylation level and nuclear translocation of FOXO1 in the hippocampus of epileptic mice, whereas EX‐527 reversed this trend. In addition, we found that FOXO1 can bind to the SLC7A11 promoter, regulating SLC7A11 transcription. The above results demonstrate that DHA exerts antiepileptic and neuroprotective effects by impeding ferroptosis through SIRT1/FOXO1/SLC7A11/GPX4 signaling pathway activation.

This study has several limitations. First, in vivo experiments employed only a single 20 mg/kg dose of DHA for intervention. While this dose effectively validated core regulatory mechanisms, its dose‐dependent effects on improving epileptic phenotypes and modulating the SIRT1/FOXO1 pathway remain unclear. Second, this study compares and analyzes the brain tissues of TBI patients and TLE patients. However, existing research has confirmed that ferroptosis plays a key role in the pathophysiology of TBI [81], which may limit the applicability of this study's conclusions. In the future, tissue around temporal lobe epilepsy foci or normal brain tissue obtained from autopsy can be used as a control group to verify these observational results. Third, we did not investigate the effects of administering DHA or EX‐527 alone on the experiment, which may increase the influence of bias factors on the results.

## 5 Conclusions

Our study demonstrated that DHA can inhibit the epileptic phenotype and improve cognitive dysfunction in epileptic mice. Moreover, DHA reduced the acetylation level of FOXO1 by activating SIRT1 expression, thereby increasing the transcriptional activity of SLC7A11, which ultimately attenuated ferroptosis in the epilepsy model. In conclusion, our study demonstrates that DHA exerts antiepileptic and neuroprotective effects by impeding ferroptosis through SIRT1/FOXO1/SLC7A11/GPX4 signaling pathway activation. These experimental results suggest that DHA has the potential to be a promising drug for the treatment of epilepsy.

## Author Contributions

Zhipeng You and Cong Huang: conceptualization, methodology, and formal analysis. Zhijie Fan and Fan Wei: data curation, writing – original draft preparation, resources and validation. Yunmin He: visualization and investigation. Shiyi Zhao and Xiaoying Gao: supervision. Jiahang Sun: writing – reviewing and editing and project administration. All authors read and approved the final manuscript.

## Funding

This work was supported by grants from the National Natural Science Foundation of China (Nos. 81871016), China Postdoctoral Science Foundation (Nos. 2018M640306), Heilongjiang Postdoctoral Grant (Nos. LBH‐Q21132 and LBH‐Q20045).

## Ethics Statement

All participants provided their informed consent prior to utilizing the study's specimens. This study was approved by the Ethics Committee of the Second Hospital of Harbin Medical University (approval number: KY2024‐036) and was conducted per the Declaration of Helsinki. Informed consent was obtained from all individual participants included in the study. All animal experiments were approved by the Ethics Committee Second Affiliated Hospital of Harbin Medical University (approval number: KY2018‐108).

## Conflicts of Interest

The authors declare no conflicts of interest.

## Supporting information


**Figure S1:** The representative trajectories of each group of mice during the probe trial.
**Figure S2:** (A‐C) Quantitative analysis of the number of Nissl‐positive cells in the CA1, CA3 and DG region of mouse brains (*n* = 3). **p < 0.01, *p < 0.05.
**Figure S3:** Representative images of DHE staining in the CA1 region. The scale bar = 50 μm.
**Figure S4:** (A) Quantitative analysis for SIRT1 cells number in the CA3 region of the hippocampus (*n* = 3). (B) Immunostaining of CA1 regions for SIRT1 after DHA injection. The scale bar = 50 μm. (C) Quantitative analysis for SIRT1 cells number in the CA1 region of the hippocampus (*n* = 3). ***p < 0.001, **p < 0.01, *p < 0.05.
**Figure S5:** Quantitative analysis of the number of Nissl‐positive cells in the CA1, CA3 and DG region of mouse brains after EX‐527 injection (*n* = 3). ***p < 0.001, **p < 0.01, *p < 0.05.
**Figure S6:** Representative images of DHE staining in the CA1 region after EX‐527 injection. The scale bar = 50 μm.
**Figure S7:** Protein expressions of SIRT1 in HT22 cells by Western Blot after EX‐527 treatment (*n* = 4). **p < 0.01, *p < 0.05.
**Table S1:** Antibody details.
**Table S2:** Sequences of qPCR primers.
**Table S3:** Clinical characteristics of TBI patients and TLE patients.
**Table S4:** Comparison of clinical characteristics between TBI patients and TLE patients.

## Data Availability

The data that support the findings of this study are available on request from the corresponding author. The data are not publicly available due to privacy or ethical restrictions.
