# Supplementary material for: Dihydroartemisinin Alleviates Neuronal Damage and Seizures in Epileptic Mice by Inhibiting Ferroptosis via the SIRT1/FOXO1/SLC7A11/GPX4 Pathway
Source: CNS Neurosci Ther. 2026 Feb 20;32(2):e70798. doi: 10.1002/cns.70798 (PMC12921727; doi:10.1002/cns.70798)
Supplement: Supplementary file 1 — Figure S1: The representative trajectories of each group of mice during the probe trial. Figure S2: (A‐C) Quantitative analysis of the number of Nissl‐positive cells in the CA1, CA3 and DG region of mouse brains (n = 3). **p < 0.01, *p < 0.05. Figure S3: Representative images of DHE staining in the CA1 region. The scale bar = 50 μm. Figure S4: (A) Quantitative analysis for SIRT1 cells number in the CA3 region of the hippocampus (n = 3). (B) Immunostaining of CA1 regions for SIRT1 after DHA injection. The scale bar = 50 μm. (C) Quantitative analysis for SIRT1 cells number in the CA1 region of the hippocampus (n = 3). ***p < 0.001, **p < 0.01, *p < 0.05. Figure S5: Quantitative analysis of the number of Nissl‐positive cells in the CA1, CA3 and DG region of mouse brains after EX‐527 injection (n = 3). ***p < 0.001, **p < 0.01, *p < 0.05. Figure S6: Representative images of DHE staining in the CA1 region after EX‐527 injection. The scale bar = 50 μm. Figure S7: Protein expressions of SIRT1 in HT22 cells by Western Blot after EX‐527 treatment (n = 4). **p < 0.01, *p < 0.05. Table S1: Antibody details. Table S2: Sequences of qPCR primers. Table S3: Clinical characteristics of TBI patients and TLE patients. Table S4: Comparison of clinical characteristics between TBI patients and TLE patients. [file CNS-32-e70798-s001.docx]

**Supplementary figures and** **figure legends**

**Fig. S1.**

**
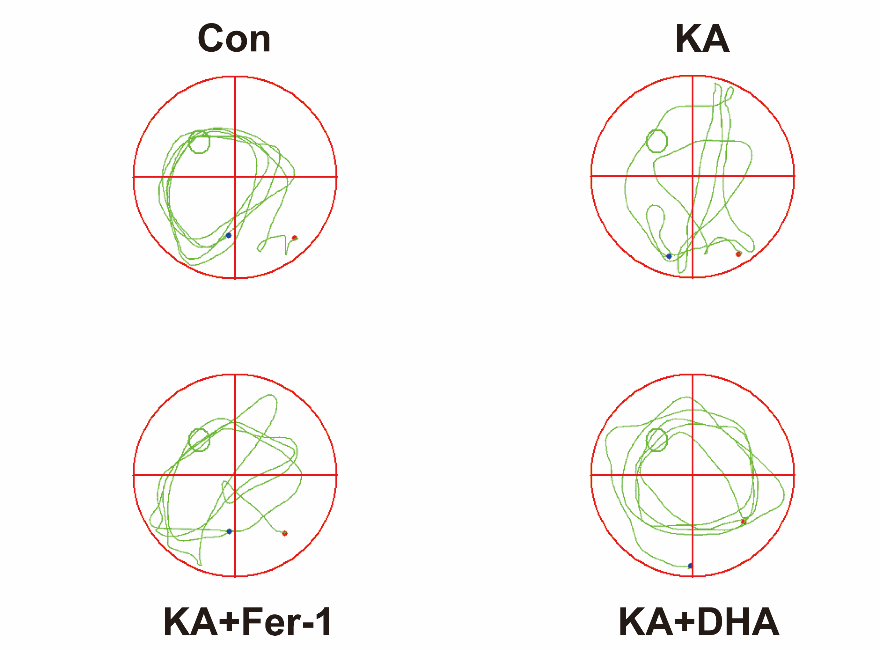
**

Fig. S1. The representative trajectories of each group of mice during the probe trial.

**Fig. S2.**

**
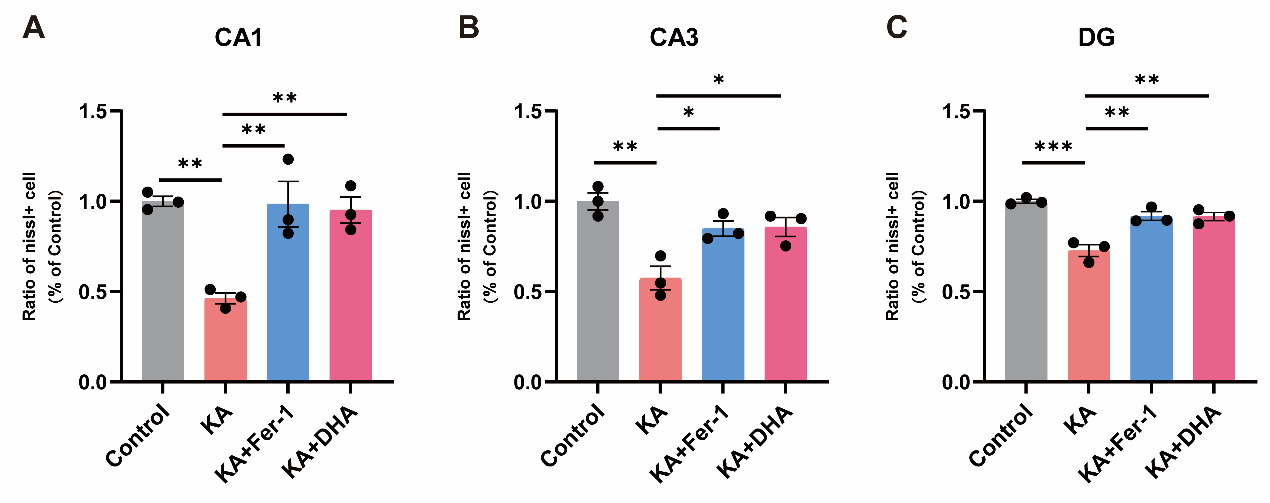
**

Fig. S2. **(A-C)** Quantitative analysis of the number of Nissl-positive cells in the CA1, CA3 and DG region of mouse brains (n = 3). ***p* < 0.01, **p* < 0.05.

**Fig. S3.**


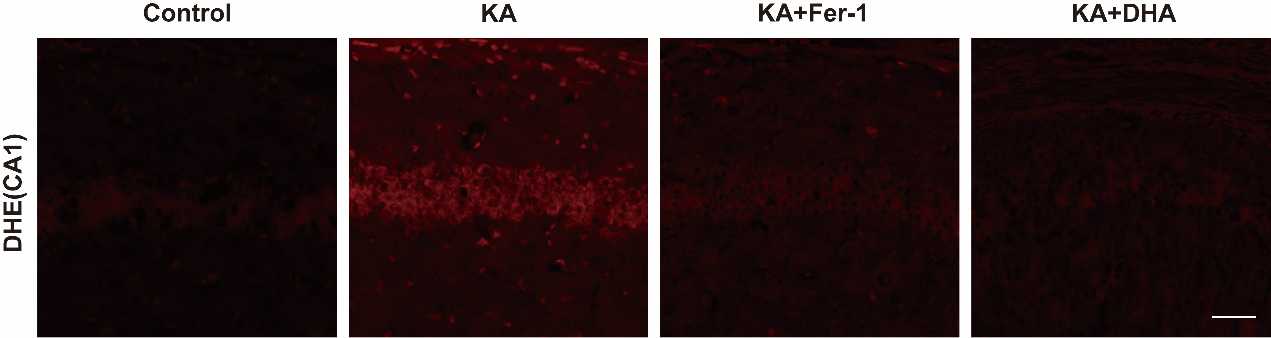


Fig. S3. Representative images of DHE staining in the CA1 region. The scale bar = 50 μm.

**Fig. S4.**

**
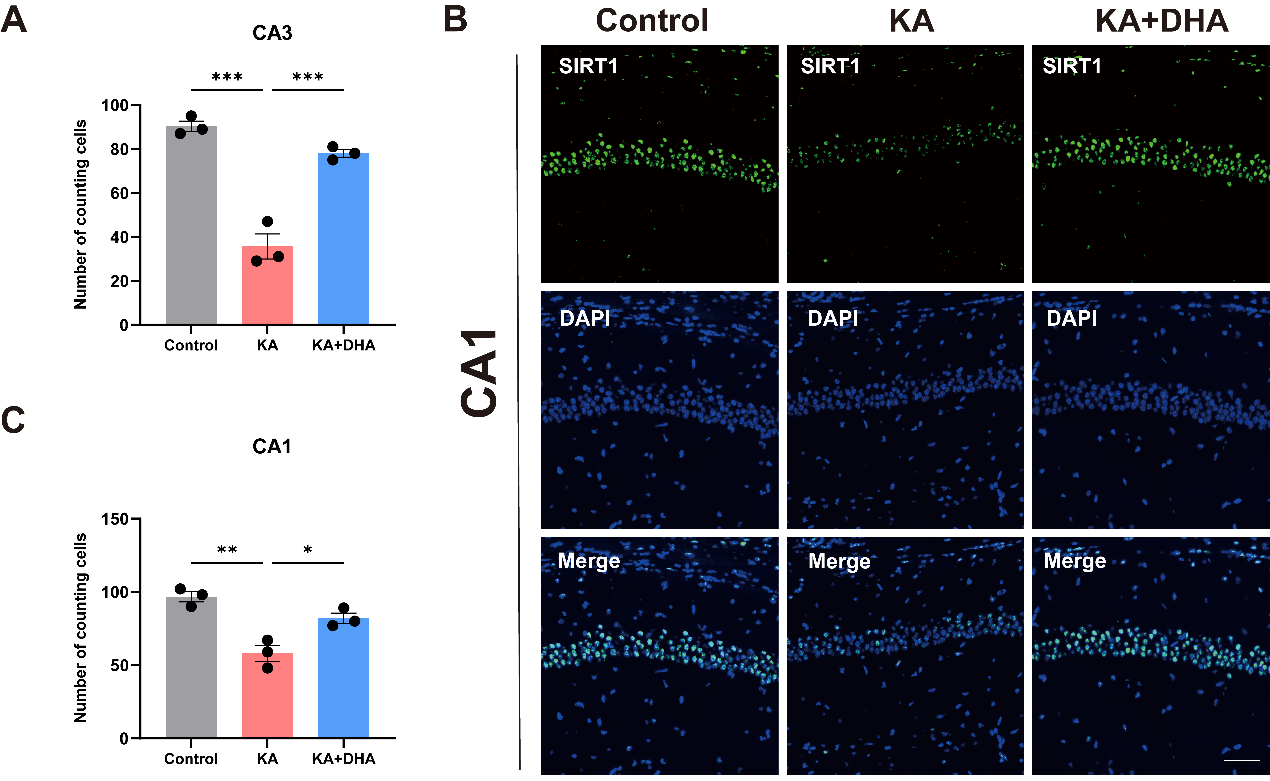
**

Fig. S4. **(A)** Quantitative analysis for SIRT1 cells number in the CA3 region of the hippocampus (n = 3). **(B)** Immunostaining of CA1 regions for SIRT1 after DHA injection. The scale bar = 50 μm. **(C)** Quantitative analysis for SIRT1 cells number in the CA1 region of the hippocampus (n = 3). ****p* < 0.001, ***p* < 0.01, **p* < 0.05.

**Fig. S5.**

**
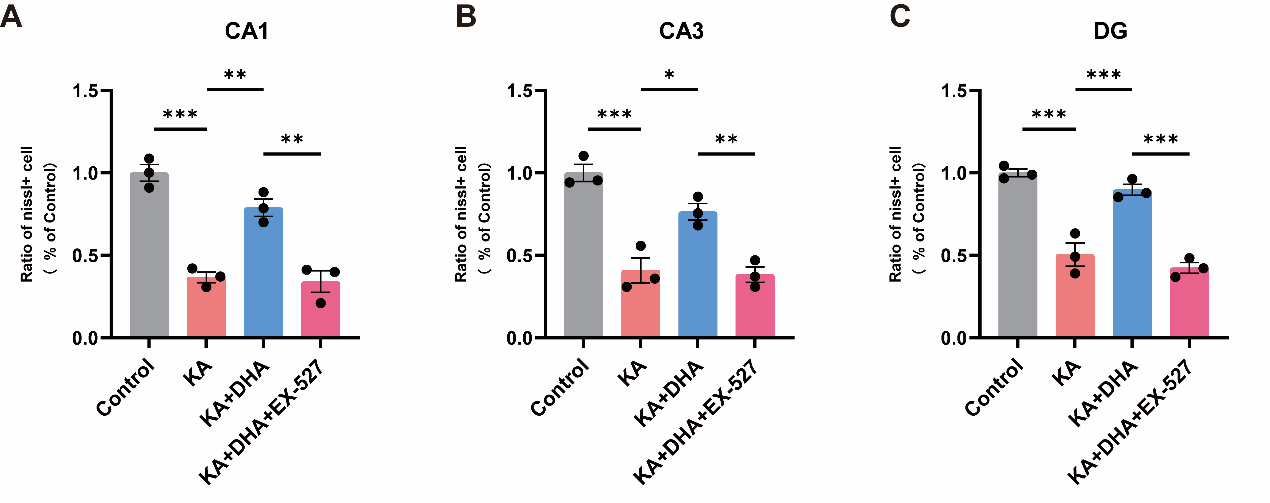
**

Fig. S5. Quantitative analysis of the number of Nissl-positive cells in the CA1, CA3 and DG region of mouse brains after EX-527 injection. (n = 3). ***p < 0.001, **p < 0.01, *p < 0.05.

**Fig. S6.**


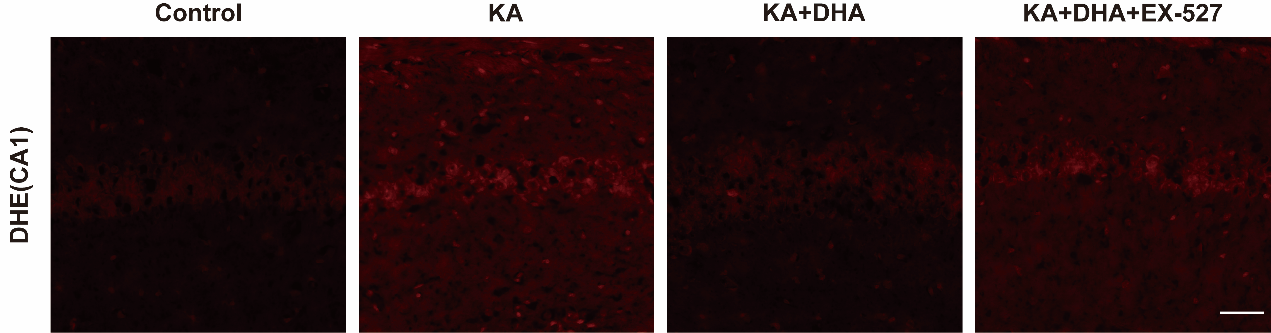


Fig. S6. Representative images of DHE staining in the CA1 region after EX-527 injection. The scale bar = 50 μm.

**Fig. S7.**

**
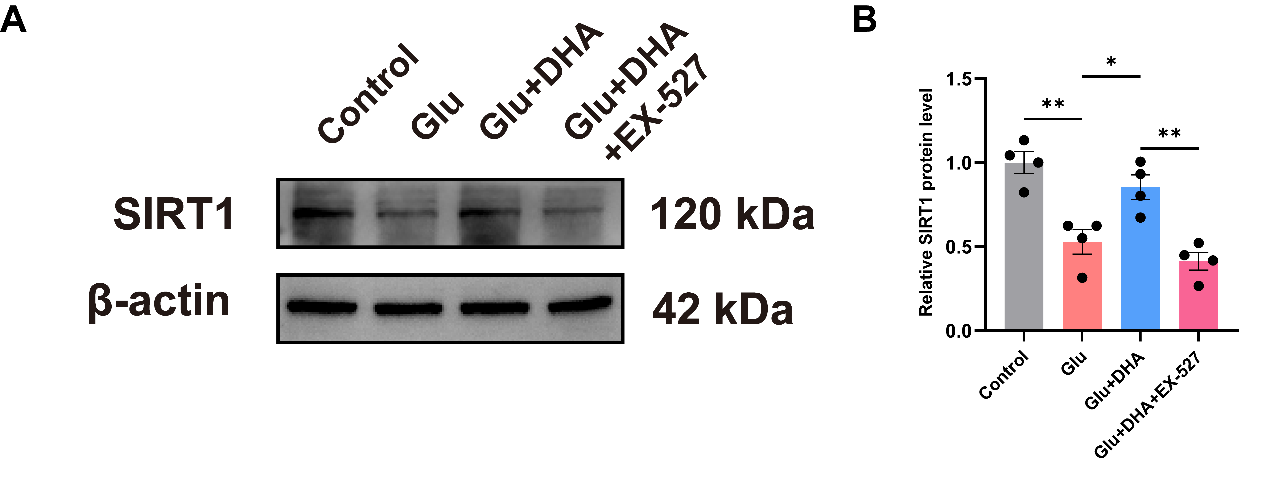
**

Fig. S7. Protein expressions of SIRT1 in HT22 cells by Western Blot after EX-527 treatment (n = 4). **p < 0.01, *p < 0.05.

**Table S1. Antibody details.**

| **Antibody** | **Source** | **Catalog Number** | **Species** | **Applications** |
| --- | --- | --- | --- | --- |
| SLC7A11 | Abmart | T57046 | Rabbit | WB (1:1000) |
| GPX4 | Proteintech | 67763-1-Ig | Mouse | WB (1:2000) |
| SIRT1 | Proteintech | 13161-1-AP | Rabbit | WB (1:2000) |
| SIRT1 | Proteintech | 13161-1-AP | Rabbit | IF (1:100) |
| FOXO1 | Proteintech | 18592-1-AP | Rabbit | WB (1:2000) |
| Ac-FOXO1 | Affinity | AF2305 | Rabbit | WB (1:2000) |
| β-actin | Proteintech | 60008-1-Ig | Mouse | WB (1:10000) |
| Histone H3 | PTMBio | PTM-6600 | Rabbit | WB (1:1000) |

**Table S2. Sequences of qPCR primers.**

| **Gene** | **Forward primer sequence** | **Reverse primer sequence** |
| --- | --- | --- |
| SLC7A11 | CCACCATCAGTGCGGAGGAG | GAAGCAGGAGAGGGCAACAAAG |
| GAPDH | GCGAAGAAAACCGCATCAC | CACACCTCACATCACCACGTC |

**Table S3. Clinical characteristics of** **TBI patients and TLE patients.**

| Cases | Sex  (M/F) | Age  (years) | disease duration(years) | medication history | Mechanism of injury |
| --- | --- | --- | --- | --- | --- |
| TBI 1 | M | 25 | 0 | None | Accident |
| TBI 2 | F | 19 | 0 | None | Fall |
| TBI 3 | F | 38 | 0 | None | Fall |
| TBI 4 | M | 39 | 0 | None | Accident |
| TBI 5 | M | 20 | 0 | None | Accident |
| TLE 1 | F | 30 | 19 | CBZ, VPA, TPM | None |
| TLE 2 | F | 32 | 10 | CBZ, VPA, TPM | None |
| TLE 3 | M | 28 | 20 | CBZ, VPA, PHT | None |
| TLE 4 | F | 19 | 11 | CBZ, VPA, PB | None |
| TLE 5 | M | 40 | 23 | CBZ, VPA, LTG | None |

M=male; F=female; CBZ, carbamazepine; PB, phenobarbital; PHT, phenytoin; VPA, valproate; LTG, lamotrigine

**Table S4. Comparison of clinical characteristics between** **TBI patients and TLE patients.**

|  | TBI group (n = 5) | TLE group (n = 5) | P values |
| --- | --- | --- | --- |
| Age (years) | 28.20 ± 9.68 | 29.80 ± 7.56 | 0.778 |
| Female/Male | 2 / 3 | 3 / 2 | 0.527 |
